# Zinc Signaling in Acute Kidney Injury

**DOI:** 10.3390/cells15111018

**Published:** 2026-06-01

**Authors:** Svetlana Lebedeva, Yan Bravyy, Anna Beknazarova, Elena A. Smolyarchuk, Kerim Mutig

**Affiliations:** 1 Scientific Center of Genetics and Life Sciences, Sirius University of Science and Technology, Sirius Federal Territory 354340, Russia; brave.yr@talantiuspeh.ru (Y.B.); beknazarova.am@talantiuspeh.ru (A.B.); 2 Department of Pharmacology, Sechenov First Moscow State Medical University, Moscow 119991, Russia; smolyarchuk_e_a@staff.sechenov.ru

**Keywords:** acute kidney injury, hypoxia, inflammation, oxidative stress, zinc

## Abstract

Acute kidney injury (AKI) is a life-threatening event prevalent in hospitalized patients but also not rare among endurance sports athletes. Hypoxia, oxidative stress, and sterile inflammation are the key pathophysiological factors driving kidney damage in AKI. Zinc is an essential trace element required for the intact function of approximately 3000 proteins (~10% of the human proteome), including over 300 enzymes for which zinc serves as a cofactor. Cell biological tasks of zinc signaling include adaptive responses to hypoxia and oxidative stress, as well as anti-inflammatory effects. The underlying molecular pathways involve modulation of hypoxia-inducible factor signaling, suppression of reactive oxygen species (ROS) generation, and inhibition of the nuclear factor kappa-light-chain-enhancer of activated B cells (NF-κB), the latter being the major pro-inflammatory transcription factor. As a catalytic cofactor for the “classical” histone deacetylases, zinc is essential for epigenetic control of gene expression, thereby exerting further adaptive effects. Apart from the intracellular zinc signaling, extracellular zinc elicits cytoprotective and anti-inflammatory effects via the G Protein-Coupled Receptor 39 (GPR39). GPR39 activation by zinc binding may exert antioxidant and anti-inflammatory effects mediated by the zinc-finger protein A20 (TNFAIP3) and NF-κB suppression, followed by reduced production of pro-inflammatory cytokines such as tumor necrosis factor (TNF), interleukin-1β (IL-1β), and IL-6. At the same time, GPR39 signaling may stimulates the release of the anti-inflammatory cytokine IL-10, thus shifting the kidney tissue towards an anti-inflammatory milieu, promoting renal recovery. The present review focuses on the role of zinc in AKI to identify potential therapeutic strategies targeting zinc signaling for renoprotection and biomarker-based risk stratification.

## 1. Introduction

### Acute Kidney Injury

Acute kidney injury (AKI) is a life-threatening condition defined as an abrupt and clinically relevant reduction in excretory kidney function, lasting up to 7 days [1,2]. According to different epidemiological estimates, the prevalence of AKI reaches 20–200 per million in the general population, 7–18% of patients in hospitals, and ~50% patients in intensive care units [3,4]. AKI is associated with high morbidity and mortality, as reflected by approximately two million AKI-related deaths per year worldwide [5]. Resolved episodes of AKI predispose to the development of chronic kidney disease (CKD) in the future, whereas partially unresolved AKI may directly transition to CKD [6]. Etiology of AKI can be divided into pre-renal, intra-renal, and post-renal causes [2]. The pre-renal conditions leading to AKI are associated with strongly reduced renal perfusion. The most frequent pre-renal AKI causes include cardiorenal syndrome, shock, abdominal compartment syndrome, kidney transplantation, and medications interfering with renal hemodynamics [2]. The intra-renal causes of AKI encompass thrombotic microangiopathies, lipid embolism, systemic infections, sepsis, drug- or toxin-induced tubular necrosis, rhabdomyolysis, contrast-induced nephropathy, acute allograft rejection, light chain cast nephropathy, and acute crystal- or metabolite-induced nephropathy (bile pigments, urate, oxalate). The post-renal AKI causes are related to acute or chronic obstruction of the urinary tract [2]. Finally, strenuous physical exercise such as ultra-endurance sports may lead to AKI originating from combined pre- and intra-renal factors, including renal vasoconstriction, dehydration with hypovolemia, rhabdomyolysis, and systemic inflammation [7].

The pathophysiological mechanisms driving AKI depend on the primary cause, but at the cellular level, they can be generalized to hypoxia, oxidative and metabolic stress, and inflammation. Hypoxia of renal tissue is the key pathophysiological event in AKI originating from insufficient oxygen delivery (renal vasoconstriction, hypotension, anemia), inappropriately high oxygen demand due to increased tubular workload, or a combination of the two mechanisms [8,9,10]. Cellular adaptations to hypoxia rely on activation of the hypoxia-inducible factors (HIFs), which are transcription factors promoting expression of over 100 genes, including erythropoietin (EPO), vascular endothelial growth factor (VEGF), glucose transporters, and glycolytic enzymes. Thus, HIF signaling stimulates erythropoiesis, angiogenesis, and anaerobic glycolysis to restore tissue homeostasis and cellular energy metabolism [8]. HIF-1 acts synergistically with the AMP-activated protein kinase (AMPK) to improve cellular energy metabolism and proteostasis via suppression of the mammalian target of rapamycin (mTOR) pathway, induction of autophagy, and reduced protein synthesis [11]. Prolonged exposure of kidneys to hypoxia, metabolic stress, or nephrotoxic drugs promotes excessive reactive oxygen species (ROS) generation, causing oxidative stress with damage to proteins, lipids, and nucleic acids, driving apoptosis or necrosis depending on the stress severity [12,13]. Stress and damage to kidney cells lead to the release of damage-associated molecular patterns (DAMPs), as well as pro-inflammatory cytokines and chemokines, thereby triggering infiltration of immune cells and sterile inflammation [14,15]. Inflammation, along with hypoxic, metabolic, and oxidative stress, promotes senescence of kidney epithelial cells with acquisition of the senescence-associated secretory phenotype (SASP) [16,17]. SASP includes pro-inflammatory cytokines such as the tumor necrosis factor (TNF), and interleukin-1 (IL-1), IL-6, and IL-18, which amplify local inflammation, suppress regeneration, and promote kidney fibrosis, thereby enhancing the risk of AKI to CKD transition [16,18].

## 2. Cellular Zinc Homeostasis

Zinc is the second most abundant trace element in the human body after iron. It serves as a cofactor for approximately 300 enzymes and represents a structural component of nearly 3000 proteins, accounting for approximately 10% of the human proteome [19]. By performing catalytic, structural or regulatory functions in various molecular signaling pathways, zinc plays a key role in cell fate and intercellular communications [20,21]. Despite relatively high intracellular zinc abundance estimated at 200–300 µM concentrations, free cytosolic zinc levels are maintained at picomolar ranges, because the dominant zinc portion is either bound to proteins or sequestered in organelles such as endoplasmic reticulum (ER), Golgi apparatus (GA), lysosomes, mitochondria, and specialized vesicles known as zincosomes [19,22,23]. Supraphysiological increases or declines in cytosolic-free Zn^2+^ are toxic. A tight regulation of cellular zinc homeostasis has evolved, including coordinated interactions between specialized zinc transporters of the SLC39A and SLC30A families along with dynamic intracellular buffering of zinc ions by metallothioneins (MTs) [19].

The SLC39A family includes 14 members, abbreviated as ZIP1 to ZIP14, standing for “zinc-regulated transporter/iron-regulated transporter” (Zrt/Irt-like protein). ZIP transporters may reside in the apical or basolateral plasma membranes, and in membranes of zinc-containing cell organelles, whereby each ZIP member displays a specific subcellular distribution pattern driven by structural features of its sorting domains. The net functional effect of ZIP-dependent transport activity is an increase in cytosolic-free Zn^2+^ concentration by import from the extracellular space and release from the intracellular zinc stores [24].

In contrast to ZIP transporters, the 10 known members of the SLC30A family synergistically act to reduce the cytosolic-free Zn^2+^ by extruding zinc ions into the extracellular space and loading them into the intracellular zinc stores. The SLC30A transporters are abbreviated as ZnT1-ZnT10, standing for “zinc transporters”. Like ZIPs, each ZnT protein is selectively assigned to certain cellular compartments, i.e., the plasma membrane or cell organelles [25].

MTs are small proteins rich in cysteine residues, forming thiolate clusters capable of binding zinc ions. MTs buffer excessive free cytosolic Zn^2+^ to maintain its levels in physiological picomolar ranges, at the same time serving as dynamic zinc stores. The bound Zn^2+^ can be mobilized to the cytosol upon oxidative stress via direct interactions between zinc-thiolates of MTs and disulfide-containing molecules such as glutathione disulfide, coenzyme A disulfide, or cystamine [26,27]. Alternatively, MTs are able to donate the bound zinc ions to a broad range of zinc-requiring proteins, including apoenzymes and zinc finger transcription factors, through a process termed direct associative ligand exchange [28]. Furthermore, lysosomal degradation of MTs leads to the liberation of zinc ions, which activate lysosomal enzymes, promoting acidification and autophagic flux [29].

**Figure 1:**
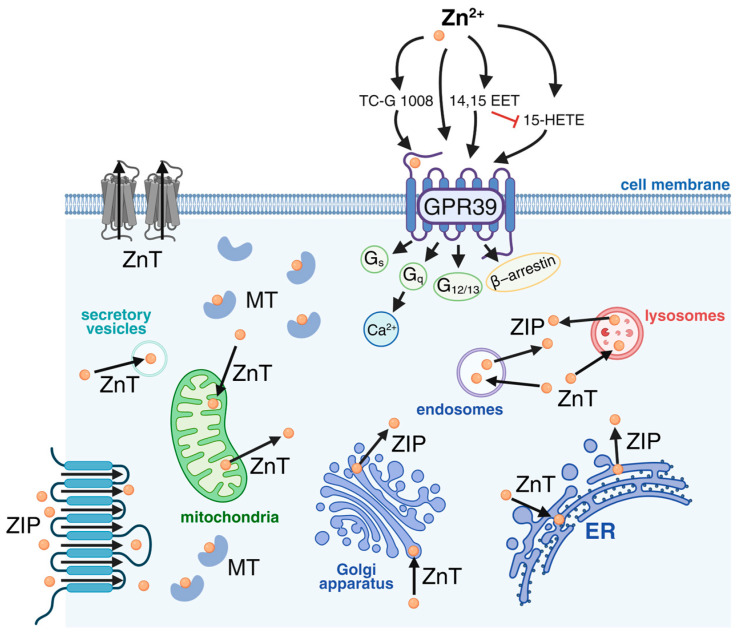
Cellular zinc homeostasis. Members of the Zrt-, Irt-like protein (ZIP) family enhance intracellular Zn^2+^ concentration by mediating the entry of extracellular zinc and releasing Zn^2+^ from intracellular compartments, primarily from the endoplasmic reticulum (ER). In contrast, members of the zinc transporter family (ZnT) reduce intracellular Zn^2+^ levels by exporting zinc ions out of the cells or loading them into intracellular storage compartments. Metallothioneins bind cytosolic Zn^2+^, thereby buffering cytosolic zinc excess. Arrows indicate the direction of Zn^2+^ transport.

Extracellular signals such as hormones, growth factors, or cytokines may lead to transient increases in cytosolic-free Zn^2+^ up to the low nanomolar range, eliciting diverse signaling events in the cell type-specific context [30]. These Zn^2+^ transients occur due to activation of ZIP transporters followed by import of extracellular zinc ions or release of intracellular Zn^2+^ from its stores. Zinc ions exert direct inhibitory effects on protein tyrosine phosphatases (PTPs), resulting in disinhibition of complementary major protein kinases, including the extracellular signal-regulated protein kinase/mitogen-activated protein kinase (ERK/MAPK), phosphatidylinositol 3-kinase/protein kinase B (PI3K/Akt), and Janus kinase/signal transducer and activator of transcription (Jak/STAT) [31,32,33]. In a cell type-specific context, activation of the aforementioned kinase pathways may induce cell proliferation, growth and survival, activation of immune cells and immune responses, or changes in gene transcription and adaptations of cell functionality [23,34]. The transient nature of the zinc waves is enabled by reciprocal activation of certain ZnTs mediating the efflux of zinc ions from the cytosol, as well as by the buffering capacity of MTs [35,36]. The coordinated functions of ZIPs, ZnTs, and MTs in response to extra- or intracellular stimuli determine the magnitude and duration of zinc waves and effects of Zn^2+^ as the second messenger (Figure 1).

Apart from the second messenger role, extracellular Zn^2+^ may function as a first messenger by binding to and activating the zinc-sensing G protein-coupled receptor 39 (GPR39) [37,38]. GPR39 exhibits significant ligand-independent constitutive activity, which is further potentiated by Zn^2+^ binding [39]. Downstream signaling may be mediated by distinct G proteins, depending on the cell type and functional context. Recruitment of Gαq/11 stimulates the Ca^2+^-dependent signaling via the phospholipase C (PLCβ) activation, generation of inositol 1,4,5-trisphosphate (IP_3_), release of Ca^2+^ from the ER, and activation of the MAPK/ERK and PI3K/Akt kinase pathways [38]. In contrast, recruitment of Gαs leads to intracellular cyclic adenosine monophosphate (cAMP) elevation and protein kinase A (PKA) activation [40]. Finally, engagement of Gα12/13 promotes the Ras homolog family member A (RhoA) and serum response element (SRE)-mediated gene expression [38,41]. The Ca^2+^-dependent signaling constitutes the dominant route for both the constitutive and Zn^2+^-stimulated GPR39 activity, whereas the cAMP-mediated signaling is undetectable constitutively and requires higher extracellular Zn^2+^ concentrations for stimulation [38,39].

In general, zinc is not only an essential functional and structural element for many proteins, but also acts as a first or second messenger eliciting major molecular signaling events mediated by Ca^2+^ or cAMP-dependent pathways.

## 3. Systemic Zinc Homeostasis

Zinc absorption takes place mainly in the duodenum and jejunum via apical ZIP4-mediated uptake and basolateral ZnT1-dependent efflux into portal circulation. Fractional absorption of dietary zinc varies between 20% and 40%, corresponding to 2–3 mg daily [42]. Zinc deficiency stimulates the zinc uptake via transcriptional upregulation of ZIP4, whereas zinc excess promotes its endocytosis, ubiquitination, and lysosomal degradation, thereby preventing further overload of the body with zinc [42]. During passage through liver sinusoids, Zn^2+^ may either bind to plasma albumin and go in the systemic circulation or enter hepatocytes via ZIP8 or ZIP14, where zinc associates with MTs for storage. Hepatocytes can excrete Zn^2+^ into bile canaliculi via apical exocytosis of vesicles loaded with zinc by ZnT2 or ZnT4. Alternatively, zinc ions can be delivered to the blood via the basolateral ZnT1 [43]. Approximately 99% of zinc is transported in the protein-bound form, whereby albumin is the main zinc carrier protein. Zinc ions bind to albumin with high affinity but moderate stability, enabling sufficient release in peripheral blood potentiated by free fatty acids, physiological or pathophysiological acidosis, and ROS during tissue inflammation [44,45]. Approximately 1% of zinc binds to transferrin. Cellular uptake of Zn^2+^ is mediated by distinct ZIP members depending on the cell type. Systemic elimination of zinc occurs mainly via feces (~50–70%), urine (~15–20%), and sweat (~10–15%) [46,47]. Notably, zinc losses with sweat may rise during prolonged strenuous exercise, increasing the risk of ensuing zinc deficiency in athletes [48,49].

## 4. Renal Zinc Handling in Health and Acute Kidney Injury

The main task of the kidneys is to filter blood plasma for the elimination of metabolic waste products, water-soluble toxins, and any excessive substances. Efficient maintenance of body homeostasis requires a high glomerular filtration rate (GFR) of approximately 90–120 mL/min with subsequent tubular reabsorption of water, electrolytes, small proteins, glucose, amino acids, and other useful substances according to the body’s needs. Under physiological conditions, the filtered Zn^2+^ undergoes nearly complete reabsorption in the proximal tubules (PTs) likely via ZIP8, ZIP10, and ZIP14 at the apical side and ZnT1 or another ZnT isoform at the basolateral side [50,51,52,53]. Participation of the distal nephron segments and collecting ducts in the urinary Zn^2+^ reabsorption is negligible under physiological conditions but a compensatory increase in the distal zinc reabsorption has been reported in response to the PT challenge by osmotic diuresis using mannitol [54]. Notably, albumin-bound zinc does not significantly contribute to urinary zinc excretion since the intact filtration barrier largely retains albumin in the plasma, while minor amounts of filtered albumin are intensively reabsorbed by PT cells. However, glomerular or tubular damage may lead to significant albuminuria and proteinuria, thereby causing substantial urinary zinc losses. Finally, like hepatocytes, renal tubular cells may be able to secrete excessive intracellular Zn^2+^ into the urine via apical exocytosis of zinc-loaded vesicles [23,25]. Overall, filtered Zn^2+^ is efficiently reabsorbed by PTs, as reflected by the low fractional excretion of zinc (FEZn) in healthy adults (FEZn~5%) [55].

Apart from the fine-tuning of renal zinc excretion, the kidney cells utilize Zn^2+^ for their own metabolic needs. The available data on the distribution of ZIP isoforms along the nephron is scarce and does not permit concrete assignments of the specific zinc transport protein to certain types of kidney epithelia. However, analysis of the available transcriptomics atlases, such as the human KPMP reference atlas, large-scale human kidney multi-omic single-cell/nucleus atlas, and canonical mouse kidney scRNA-seq atlas, is suggestive of the expression of multiple ZIP and ZnT isoforms in kidney epithelia [56,57,58,59].

**Figure 2:**
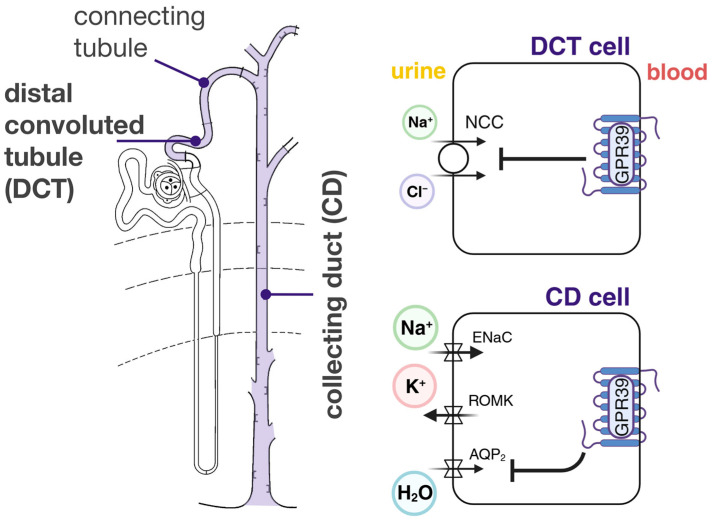
Renal distribution and function of GPR39. The left panel demonstrates sites of GPR39 expression in the nephron, including the distal convoluted tubule (DCT), and principal cells of the connecting tubule (CNT) and collecting duct (CD). The panel on the right describes the functional effects of GPR39 activation. GPR39 exerts inhibitory effects on the Na^+^,Cl^−^-cotransporter (NCC) in DCT. Increased Na^+^ load in the ensuing CNT and CD drives the Na+ reabsorption via the epithelial sodium channel (ENaC), paralleled by K^+^ secretion via the renal outer medullary channel (ROMK). GPR39 further inhibits aquaporin-2 (AQP2)-dependent water reabsorption in CNT and CD.

Finally, plasma Zn^2+^ levels affect renal function via GPR39, which is present in the distal nephron and collecting duct epithelia [60]. A recent study in wild-type and GPR39-deficient mice showed that renal GPR39 activation reduces urinary concentration, in part by interfering with the effects of vasopressin [60]. Genetic deletion and pharmacological activation of GPR39 suggest that this receptor opposes vasopressin-induced salt reabsorption in the distal convoluted tubule and water reabsorption in the collecting duct (Figure 2) [60,61].

AKI is frequently associated with PT damage due to high metabolic demand and vulnerability of PT cells [62]. Depending on the AKI etiology, acute tubular injury (ATI) or necrosis (ATN) may result from ischemia, nephrotoxicity, or a combination of both [2,63]. PTs are the main site of renal zinc reabsorption, which may explain increased urinary zinc excretion in AKI patients reported by several studies [64,65]. In this context, urinary zinc has been discussed as an early AKI biomarker [64,65]. Thus, urinary zinc may be considered for biomarker panels assessing renal stress in urine and blood samples. However, the available evidence regarding the utility of urinary Zn^2+^ as a reliable AKI biomarker is limited and requires further investigation. Apart from the urinary zinc wasting, local dysregulation of zinc homeostasis appears to contribute to the renal tubular damage during AKI [66]. The latter has been linked to ZIP8, which is highly abundant in PT cells and mediates the influx of Zn^2+^ and some other bivalent metals, including Fe^2+^, Mn^2+^, Cd^2+^, and Co^2+^ [51,66,67,68,69,70]. A rare polymorphism in the SLC39A8 gene (rs13107325 SNP) encoding for ZIP8 has been associated with diminished renal zinc levels and susceptibility to kidney injury in experimental AKI and CKD models [66]. Therefore, reduced ZIP8-mediated Zn^2+^ uptake leads to intracellular zinc deficit, predisposing to kidney damage. Along the same line, reduced cytosolic Zn^2+^ levels have been reported in the cerebro-renal syndrome (Birk–Landau–Perez syndrome) caused by loss-of-function mutations in the SLC30A9 gene encoding ZnT9 [71]. Increased systemic and cellular Zn^2+^ availability has been associated with improved AKI outcomes in experimental studies [72]. Therefore, intact cellular zinc homeostasis is critical for kidney health, whereas both zinc deficiency and excess increase the renal susceptibility to AKI and CKD.

## 5. Zinc Deficiency and Acute Kidney Injury

Zinc is an essential component in cellular management of oxidative and nitrosative stress [73,74,75]. The strength of zinc binding to MTs is highly sensitive to changes in cellular redox state. Oxidation of sulfhydryl groups of MTs leads to Zn^2+^ release, so that a shift to oxidizing conditions leads to increased availability of free cytosolic zinc ions, whereas a shift to a reducing environment promotes binding of zinc ions with MTs [73]. Moreover, MTs have been increasingly recognized as potent electrophilic scavengers and cytoprotective agents against oxidative/nitrosative stress [76,77]. Therefore, oxidative/nitrosative stress leads to transient increases in cytosolic Zn^2+^, which then acts as a second messenger at multiple levels with the net antioxidant effect [73]. At the level of mitochondria, zinc ions may compete with calcium for import, thereby preventing mitochondrial calcium overload with ensuing stabilization of electron transport chain integrity and reduction in superoxide generation, although zinc excess may inhibit the mitochondrial respiratory function instead [78,79,80]. Furthermore, zinc ions suppress superoxide generation by inhibiting assembly of nicotinamide adenine dinucleotide phosphate (NADPH) oxidases and suppressing their activation [81,82]. Finally, zinc is a structural element of the superoxide dismutase (SOD), which converts superoxide into hydrogen peroxide. Adequate cytosolic Zn^2+^ availability may prevent SOD misfolding and support its catalytic activity [83]. Therefore, several complementary mechanisms may underlie the antioxidant effects of zinc. These mechanisms may also be nephroprotective by myoglobin-induced oxidative stress during rhabdomyolysis, which frequently occurs in endurance athletes. Release of myoglobin into circulation due to exertional muscle damage has been associated with induction of oxidative stress in kidney epithelia and renal vasoconstriction, whereas adequate intracellular Zn^2+^ content may reduce nephrotoxicity [84,85].

Apart from SOD, zinc serves as a critical cofactor in the catalytic active site of “classical” histone deacetylases (HDACs) [86]. HDACs remove acetyl groups from histones with functional consequences for chromatin remodeling and gene expression profiles [87]. The binding of acetyl groups to histone lysine residues mediated by histone acetyltransferases (HATs) reduces their positive charge, leading to decreased interactions of histones with DNA, less compact chromatin structure, improved accessibility of DNA to RNA polymerases, and facilitated gene expression. Consequently, histone deacetylation by HDACs promotes interactions between histones and DNA, resulting in gene repression [86,87,88]. Thus, posttranslational modifications of histones by HATs and HDACs exert epigenetic control upon eukaryotic gene transcription [87]. Eighteen human HDACs are grouped into four classes (classes I–IV). The classes I, II, and IV, comprising eleven enzymes, belong to the “classical” zinc-dependent HDACs, whereas the class III encompasses NAD+ dependent sirtuins [86,87]. Class I HDACs display predominant nuclear localization, whereas class II and IV HDACs readily translocate between the nucleus and cytosol. In the latter, HDACs have been implicated in the deacetylation of a wide range of non-histone proteins playing distinct roles in metabolism, redox homeostasis, actin cytoskeleton remodeling, and ion transport [89,90,91,92]. Dysregulation of HDACs has been associated with cancer, which prompted the development of HDAC inhibitors as anti-cancer therapeutic strategies [93,94]. Apart from suppression of certain tumor types, HDAC inhibitors reduce inflammation and fibrosis, thus showing potential for repurposing towards treatment of cardiovascular and renal diseases [95,96]. Renoprotective effects of HDAC inhibitors have been supported by a growing body of experimental evidence obtained in rodent models [97,98]. Mechanistically, HDACs may interfere with the autophagy pathway while promoting the transforming growth factor (TGF-β) signaling [97,98,99]. In this context, HDAC inhibitors may enhance autophagy, suppress apoptosis of kidney epithelia, and retard renal fibrosis [96,98]. Modeling of AKI in mice using renal ischemia–reperfusion injury (IRI) revealed dynamic responses of several HDAC isoforms in kidney epithelial, vascular, and interstitial cells [100]. Altered expression profiles of HDACs were detected in other AKI models as well [101,102]. Pan-HDAC and selective class I HDAC inhibitors exerted potent anti-fibrotic effects in rodent models of kidney damage due to suppression of the TGF-β signaling [99,103,104]. In contrast, experiments in zebrafish suggested that activities of HDAC2, HDAC6, and HDAC8 may be required for repair of kidney tissue during the AKI resolution phase, and these effects are partly mediated by NF- κB [105]. Thus, the roles of distinct HDAC isoforms in AKI may be model- and context-dependent, requiring further investigation for improved translation of HDAC inhibitors to nephrological applications. Finally, studies in genetically engineered mice and cultured cells suggested a role for HIF-1 acetylation in the regulation of its activity [106]. Zinc-dependent HDAC isoforms have been shown to facilitate the HIF-1 function in diverse cell culture models, mostly in the context of cancer research [107]. Although both HDACs and HIFs exert a strong impact on the AKI course, their functional interconnections still remain to be clarified [108,109].

**Figure 3:**
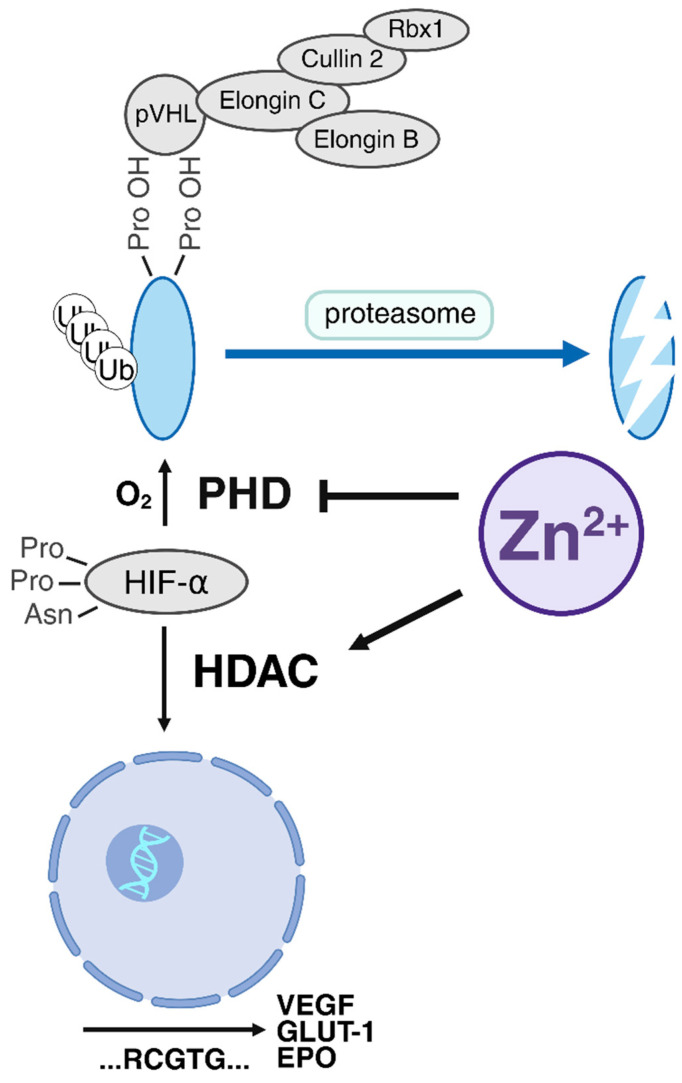
Effects of intracellular zinc availability on the cellular response to hypoxia. Cytosolic zinc exerts inhibitory effects on prolyl hydroxylase domain-containing proteins 2 and 3 (PHD2/3), which are the key oxygen-sensing enzymes promoting degradation of hypoxia-inducible factor alpha subunits (HIF-α) in normoxia. Mobilization of free cytosolic Zn^2+^ increases HIF-α stability and enhances adaptations to hypoxia at the cellular and tissue levels. Zinc also serves as a catalytic cofactor for histone deacetylases (HDAC), which stabilize HIF-α, thereby facilitating the HIF-signaling. …RCGTG… indicates the HRE motif mediating HIF-dependent gene expression. Arrows indicate positive regulation or downstream signaling, whereas blunt-ended lines indicate inhibition.

Administration of ZnCl_2_ to rats prior to renal IRI exerted renoprotective effects due to stimulation of HIF-1α and HIF-2α [110]. Zn^2+^ may inhibit the catalytic activity of the prolyl hydroxylase 2 (PHD2) due to the presence of DEAF1 (MYND)-type zinc finger domain in its N-terminus [111]. Since PHD2 promotes degradation of HIF-1 α and HIF-2 α, suppression of its activity by zinc would increase cellular abundance of these HIF isoforms. Moreover, Zn^2+^ has been shown to inhibit PHD3 activity as well [112]. The stabilizing effects of Zn^2+^ on HIF-isoforms are multifactorial and largely non-competitive with Fe^2+^ at the active PHD2 or PHD3 sites (Figure 3) [113].

In contrast, treatment of human prostate cancer and glioblastoma cells with ZnCl_2_ inhibited HIF-1α via inducing its proteasomal degradation [114]. Since ZnCl_2_ was administered at a high and potentially cytotoxic dose (100 µM), the observed effects may reflect cytotoxicity rather than cell biology [115]. Generally, cell culture studies on the effects of intracellular zinc on HIF-1α show heterogeneous results, which are likely attributable to different cell lines, zinc supplementation doses, and protocols. Induction of zinc-deficiency in human microvascular endothelial cells (HMVEC) by zinc chelation promoted nuclear translocation of HIF-1α with ensuing endothelin-1 secretion [116]. Along the same line, another study showed that cytosolic zinc may stabilize HIF-1α but suppresses the nuclear translocation of the aryl hydrocarbon receptor nuclear transporter (HIF-1β) via induction of a truncated, dominant-negative HIF-1α isoform [117]. Further studies suggest that disruption of zinc homeostasis interferes with the HIF-1α expression, whereas excessive intracellular Zn^2+^ levels exert cytotoxic effects partly mediated by HIF-1α overexpression [118,119]. In view of the contradictory previous results and multiple roles of intracellular zinc, further studies are mandatory to substantiate the information on the effects of cytosolic Zn^2+^ on HIF isoforms and their downstream signaling in the context of AKI.

Both hypoxia and oxidative/nitrosative stress trigger DAMPs release with ensuing sterile inflammation amplifying the kidney damage [120]. Cytosolic Zn^2+^ exerts potent anti-inflammatory effects by inhibition of the NF-κB signaling at multiple levels. Monocytes treated with zinc exhibited blunted TNF production in response to lipopolysaccharide (LPS) due to zinc-dependent inhibition of the IκB kinase (IKK) upstream of NF-κB [121]. Likewise, the tumor necrosis factor alpha-induced protein 3 (TNFAIP3 or A20), a zinc-finger protein, has been implicated in Zn^2+^-dependent suppression of IKKα/NF-κB signaling with the ensuing reduction in TNF and IL-1 production [122]. Notably, NF-κB signaling elicits negative feedback via promoting ZIP8 expression and ZIP8-mediated Zn^2+^ influx to prevent excessive inflammatory reaction [123]. In contrast, depletion of cytosolic zinc by chelating agents is associated with enhanced NF-κB activity and expression of the downstream inflammatory genes, including TNF and IL-1, suggesting that zinc deficiency provokes inflammation [124]. Zinc deficiency has further been linked with activation of the NACHT, LRR, and PYD domains-containing protein 3 (NLRP3) inflammasomes, driving the processing and secretion of IL-1 in macrophages [125]. From another perspective, zinc-dependent stimulation of IL-1 production by macrophages and monocytes has been reported in rheumatoid arthritis (RA), suggesting a permissive role of Zn^2+^ in physiological vs. pathophysiological conditions [126]. Increased expression of ZIP8 (zinc importer) in monocytes and macrophages of RA patients may support their sustained activation and IL-1 production in the context of autoimmunity, since zinc is critical to the execution of virtually all immune responses [126].

Apart from the intracellular zinc-induced reactions, extracellular zinc may potentially affect the AKI dynamics via GPR39. To our knowledge, no studies have examined the role of GPR39 activity in the pathophysiology of AKI until now. Experimental studies in GPR39-deficient mice suggest that GPR39 may antagonize the effects of vasopressin, thereby promoting urine production [60,61]. Notably, polyuria frequently accompanies the AKI resolution stage [127,128,129,130]. However, the role of GPR39 herein is debatable since plasma Zn^2+^ levels are rather low in AKI patients [131,132]. Thus, the polyuria commonly occurring during the AKI recovery is likely associated with tubular dysfunction [127,128,129,130]. Apart from modulation of vasopressin action in the kidney, GPR39 has been associated with renal effects of angiotensin II (AngII) [133]. Chronic AngII infusion enhanced the renal GPR39 expression along with the induction of renal fibrosis in mice, whereas genetic deletion of GPR39 alleviated the AngII-induced kidney damage, suggesting that GPR39 antagonism may be instrumental in chronic kidney diseases [133]. The underlying molecular mechanisms involve modulation of the ribonucleotide reductase M2 (RRM2) activity, which is relevant to the regulation of tubular ferroptosis [133,134]. Further studies utilizing GPR39 antagonists are necessary to clarify their renal effects in acute and chronic kidney pathologies.

In a more global context, GPR39 may mediate anti-inflammatory effects of extracellular Zn^2+^, which may be relevant during AKI resolution [135]. In this context, zinc supplementation has been shown to improve the AKI prognosis in critically ill patients [131]. Recent studies suggest that GPR39 exerts anti-inflammatory effects at multiple levels, including induction of A20 and suppression of NF-κB [136]. GPR39 has further been shown to increase production of IL-10 by macrophages [137]. IL-10 is a potent anti-inflammatory cytokine with well-documented renoprotective properties. IL-10 has been shown to improve outcomes in animal models of ischemia–reperfusion injury closely reflecting human AKI pathophysiology [138]. IL-10 further decreased the degree of renal fibrosis in a mouse model of unilateral ureteral obstruction (UUO), whereas mice with genetic IL-10 deletion developed a more severe phenotype compared to wild-type controls. Favorable effects of IL-10 were mediated by decreasing endoplasmic reticulum stress and inflammation [139,140]. Therefore, both intracellular and extracellular Zn^2+^ may exert synergistic anti-inflammatory effects. Further experimental studies are mandatory for the clear dissection between the two signaling pathways and improved understanding of their molecular details.

Zinc deficiency has been increasingly recognized as an independent risk factor for AKI development in CKD patients [132]. Furthermore, zinc sulfate supplementation was associated with improved survival in critically ill patients with AKI [131]. By analogy with patients, zinc deficiency may exert a negative impact on renal outcomes in athletes, especially in endurance sports with a relatively high incidence of AKI after intensive training or competitions [7]. Imbalanced nutritional strategies, along with sustained intensive training, predispose athletes to a relative or absolute zinc deficiency [141,142]. Thus, insufficient intracellular or extracellular zinc availability may potentially affect the risk and outcomes of AKI caused by strenuous exercise.

## 6. Translational Perspectives

Zinc deficiency has been increasingly recognized as an independent risk factor provoking or aggravating both AKI and CKD [132,143]. The impact of zinc supplementation in adult and pediatric CKD patients was addressed in numerous studies, whereas the respective effects in AKI patients received only minor attention so far [131,132,144,145]. A recent meta-analysis of 23 randomized controlled clinical trials (RCTs) on the effects of zinc supplements in adult CKD patients suggested general benefits, including increases in body weight, serum zinc concentrations, and serum albumin levels [146]. However, the authors of the meta-analysis explicitly stress the low quality of the available evidence, appealing for further studies in this direction [146]. An older meta-analysis of 15 RCTs on the effects of zinc supplementation in hemodialysis patients revealed benefits for their nutritional status, along with decreases in serum C-reactive protein (CRP) and malondialdehyde levels, suggesting a relief of inflammation and oxidative stress [147]. While the general benefits of correcting zinc deficiency in nephrological patients are obvious, validated protocols providing exact information on the supplementation form, dose, and duration need to be established.

Several inorganic and organic formulations of peroral zinc supplements have been tested clinically (Table 1). The reported adverse effects are moderate and mainly affect the gastrointestinal tract, for instance, nausea, gastric irritation, or constipation. The organic peroral zinc supplements, such as amino acid chelates, demonstrate higher bioavailability and better tolerance compared to inorganic zinc formulations (Table 1). Apart from zinc amino acid chelates, several zinc complexes of imidazole derivatives showed promising results in experimental studies but their clinical validation is pending [148,149]. Parenteral zinc supplements are available as well (Table 1). Zinc sulfate or zinc chloride solutions may be used as part of total parenteral nutrition to prevent or cure zinc deficiency. Generally, parenteral zinc formulations show better bioavailability, faster correction of zinc-deficient states, and milder side effect profiles compared to peroral zinc supplements [150,151,152]. It is tempting to speculate that parenteral or organic peroral zinc supplements bear nephroprotective potential in AKI patients, but effects may depend on the patient’s zinc status, i.e., zinc deficiency or adequate zinc metabolism. Moreover, zinc excess can exert nephrotoxic effects, provoking or aggravating AKI [153]. According to the Food and Drug Administration (FDA, USA) or European Food Safety Authority (EFSA, EU) guidelines, the maximal daily peroral zinc intake in adults should not exceed 40 mg/day or 25 mg/day, respectively [154]. The recommended daily doses vary depending on the country and are higher for men than for women [154]. Intake of high zinc doses may induce acute or chronic renal damage, including hematuria, proteinuria, tubular injury, interstitial nephritis, or copper deficiency [153,154,155,156]. Further studies are mandatory to define the optimal zinc formulations, doses, and administration routes in AKI patients. Zinc supplementation protocols should consider the body zinc status and the kidney function of nephrological patients.

Rapid enhancement of intracellular Zn^2+^ signaling can be potentially reached by zinc ionophores such as pyrithione [169]. Pyrithione has shown antiviral, immunomodulatory, and anticancer activities in experimental studies [170,171]. However, the therapeutic potential of zinc ionophores has not been studied in AKI or CKD models. Moreover, these drugs are not exclusively zinc-specific, permitting transport of other bivalent metals. Cytotoxicity is a further critical factor limiting clinical use of zinc ionophores [170,171].

In general, intracellular Zn^2+^ availability is critical to adequate cellular responses to hypoxia, oxidative stress, and inflammation, i.e., to the principal pathophysiological AKI triggers [2,132]. The molecular mechanisms mediating adaptive effects of zinc signaling include stimulation of HIFs, improvement of mitochondrial functions, and suppression of pro-inflammatory cytokines. These mechanisms may also be relevant to exercise-associated AKI.

Apart from multiple tasks of intracellular zinc in cellular metabolism, extracellular GPR39 signaling emerges as a potential renoprotective target. While balanced nutrition with adequate zinc consumption reduces the risk of kidney diseases, pharmacological modulation of GPR39 signaling may be instrumental in AKI due to anti-inflammatory potential revealed by experimental studies [38,135,172].

In summary, monitoring and correcting zinc status may decrease the incidence and severity of AKI in hospitalized patients. Endurance athletes may represent another population at increased AKI risk [7]. Subclinical zinc deficiency is common in endurance athletes due to specific dietary requirements and intensive training schedules [142]. Future studies will promote the development of personalized nephroprotective protocols for zinc-related risk stratification, considering the individual zinc status and renal function.

## Figures and Tables

**Table 1:** Comparative characteristics of validated zinc formulations.

*Inorganic Zinc Supplements for Parenteral Use (Injections, Total Parenteral Nutrition)*				
Formulation	Bioavailability	Tolerability	Indications	References
Zinc sulfate (i.v. infusions)	++++	++++	Zinc deficiency	[150,152]
Zinc chloride (i.v. infusions)	++++	+++	Zinc deficiency	[152]
*Inorganic zinc supplements for peroral use (tablets, capsules)*				
Formulation	Bioavailability	Tolerability	Indications	References
Zinc picolinate	+++	++++; GI	Zinc deficiency	[157,158]
Zinc citrate	+++	+++; GI	Zinc deficiency	[157,158]
Zinc acetate	++	+++; GI	Wilson’s disease	[159]
Zinc sulfate	++	++; GI complaints	Zinc deficiency, childhood diarrhea	[160,161]
Zinc oxide	++	+++; GI complaints	Multivitamins, low-cost supplements	[157]
*Organic zinc supplements for peroral use*				
Formulation	Bioavailability	Tolerability	Indications	References
Zinc gluconate	+++	++++; GI	Zinc deficiency, common cold	[157,162]
Zinc amino acid chelates	+++	++++	Zinc deficiency	[163,164], NCT01791608 [165]
Zinc (bis)glycinate	++++	++++; GI	Zinc deficiency, long-term supplementation, and sensitive GI patients	[157,166,167]
Zinc-histidine	+++	++++	Zinc deficiency	[168]

Table 1 legend. Reported data on bioavailability and tolerability of different zinc formulations are summarized and presented semiquantitatively: excellent (++++), high (+++) and moderate (++). GI—gastrointestinal complaints. References are provided in brackets.

## Data Availability

No new data were created or analyzed in this study.

## Abbreviations

The following abbreviations are used in this manuscript:

AKI	Acute kidney injury
AMPK	AMP-activated protein kinase
AQP2	Aquaporin-2
ATI	Acute tubular injury
ATN	Acute tubular necrosis
CD	Collecting duct
CKD	Chronic kidney disease
CNT	Connecting tubule
DAMPs	Damage-associated molecular patterns
DCT	Distal convoluted tubule
ENaC	Epithelial sodium channel
EPO	Erythropoietin
ERK	Extracellular signal-regulated kinase
FEZn	Fractional excretion of zinc
GA	Golgi apparatus
GFR	Glomerular filtration rate
GPR39	G protein-coupled receptor 39
HATs	Histone acetyltransferases
HDAC	Histone deacetylase
HIFs	Hypoxia-inducible factors
LRR	Leucine-rich repeat
MAPK	Mitogen-activated protein kinase
mTOR	Mechanistic target of rapamycin
MTs	Metallothioneins
NACHT	NAIP, CIITA, HET-E, and TP1 domain
PHD2	Prolyl hydroxylase domain-containing protein 2
PTPs	Protein tyrosine phosphatases
PTs	Proximal tubules
PYD	Pyrin domain
ROMK	Renal outer medullary potassium channel
ROS	Reactive oxygen species
SASP	Senescence-associated secretory phenotype
SOD	Superoxide dismutase
STAT	Signal transducer and activator of transcription
TNF	Tumor necrosis factor
VEGF	Vascular endothelial growth factor
